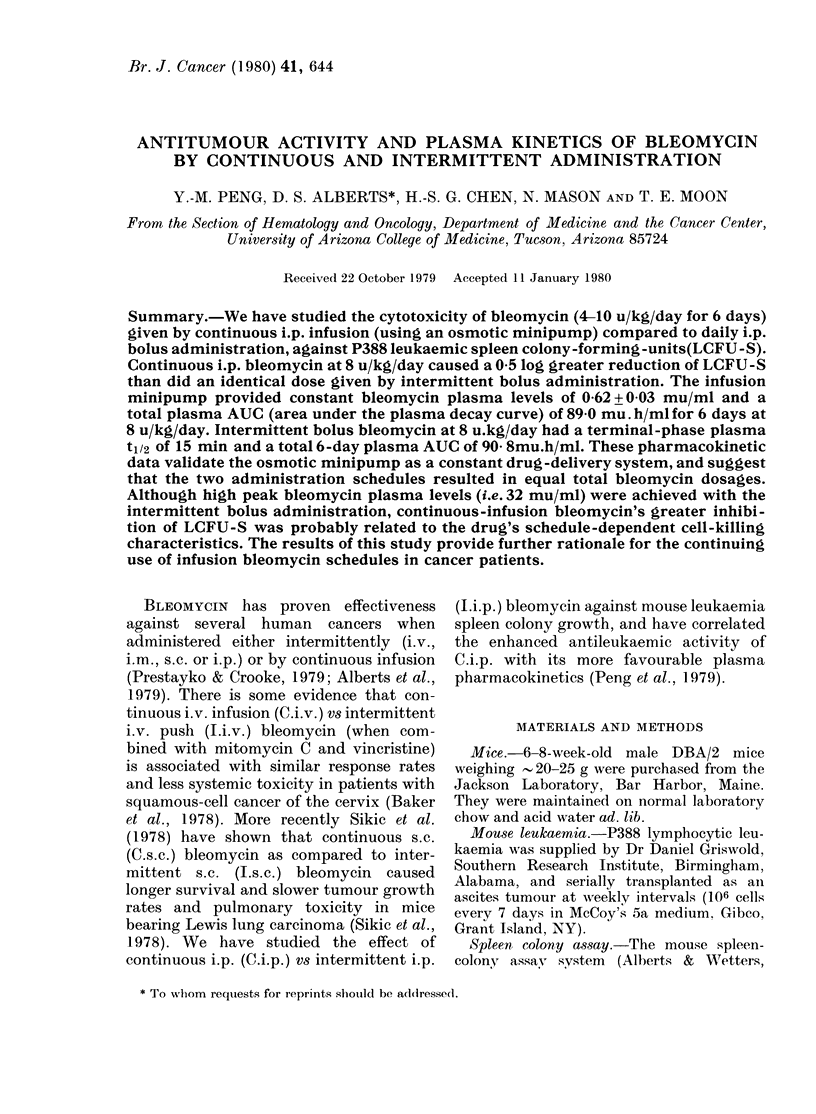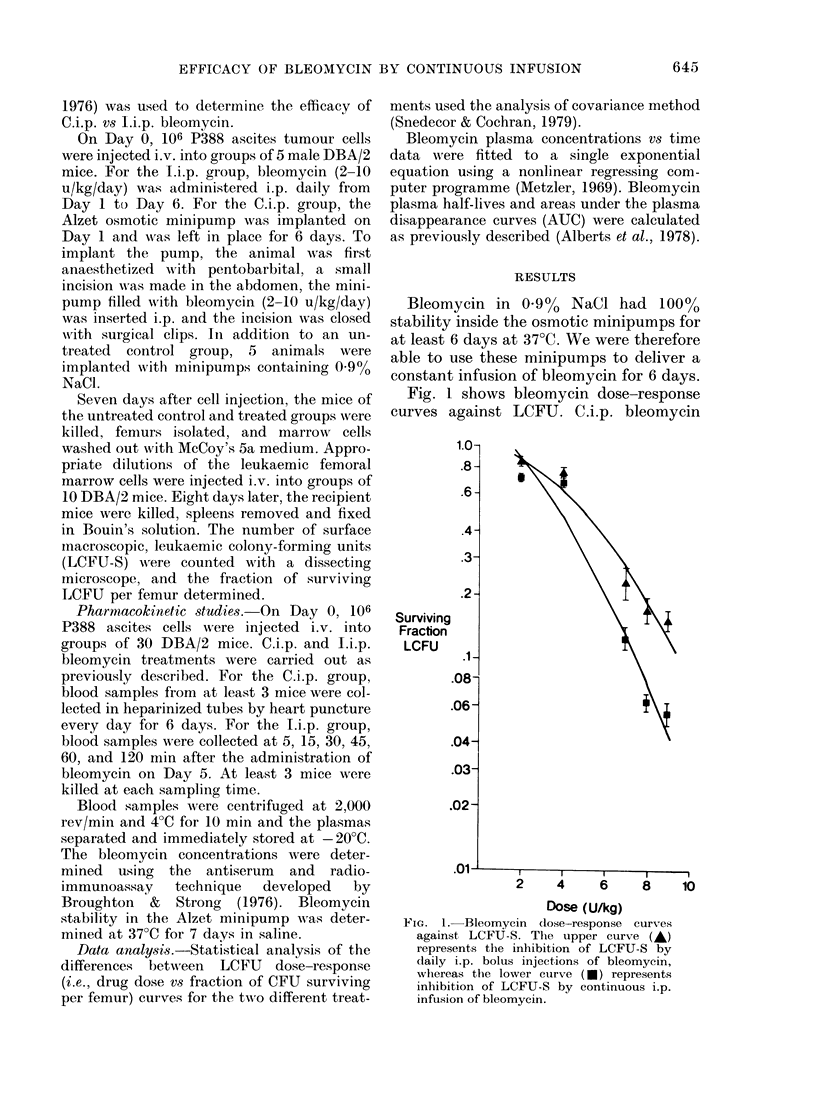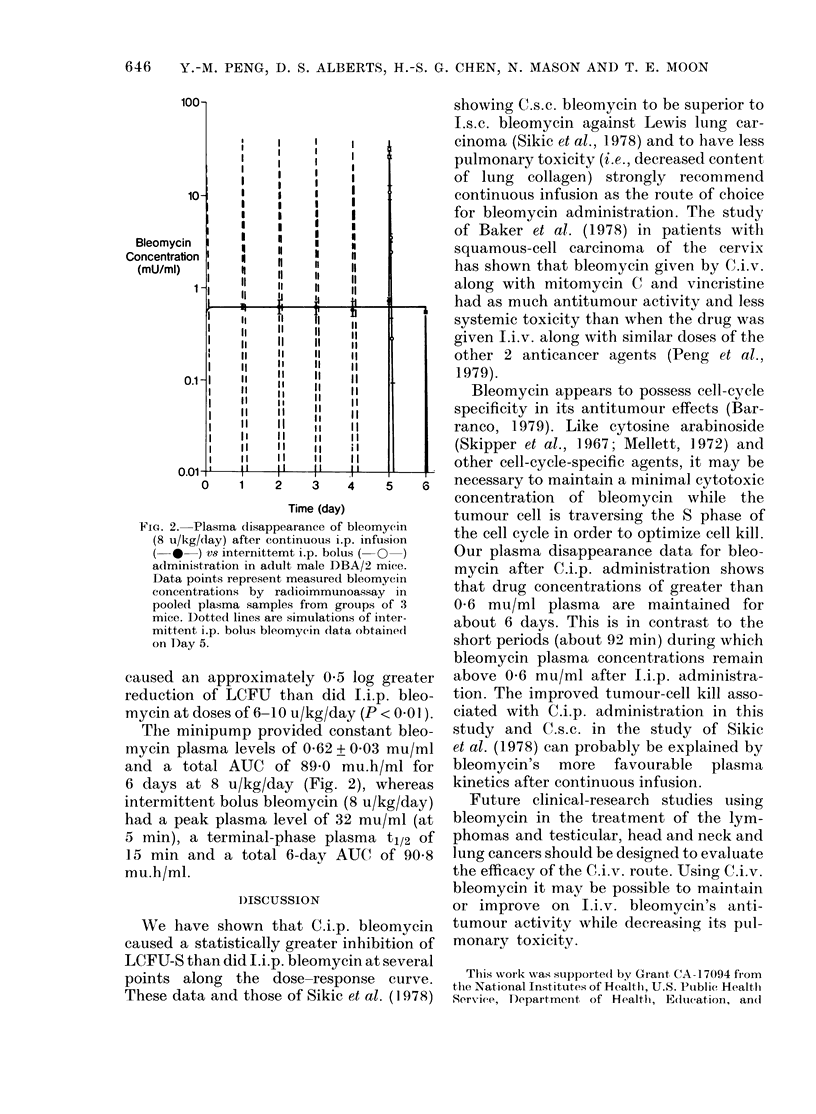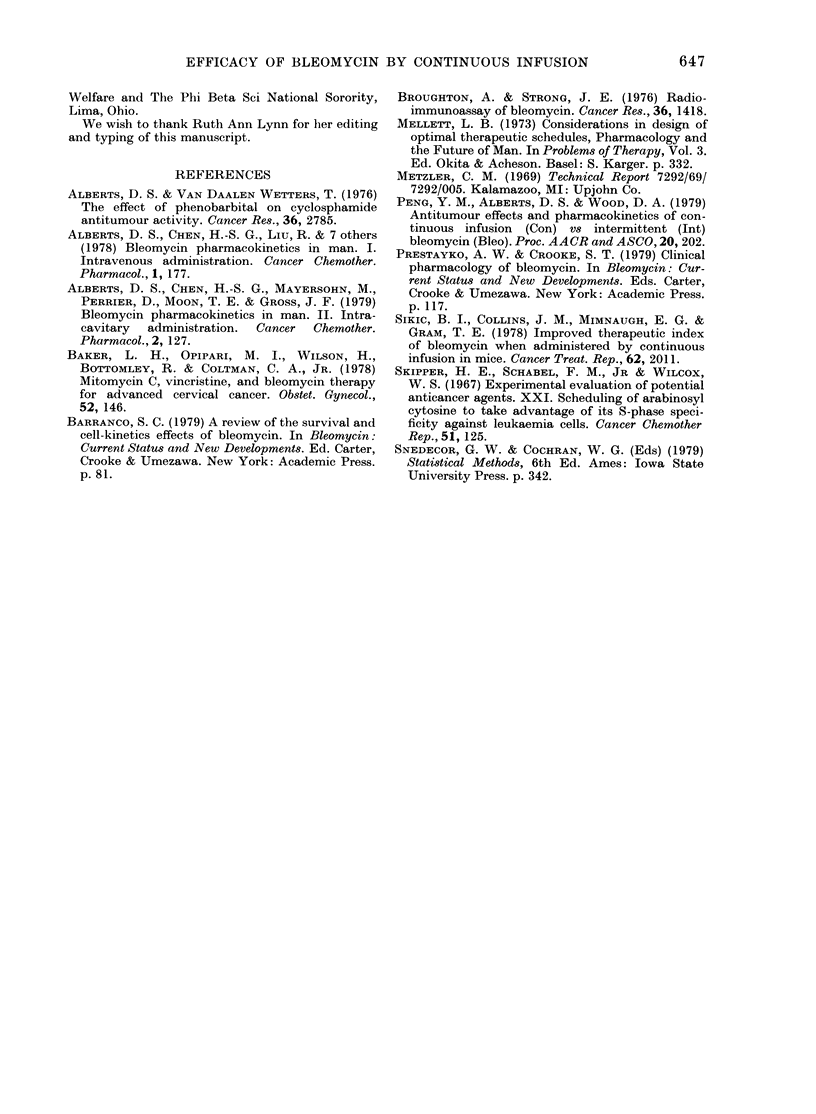# Antitumour activity and plasma kinetics of bleomycin by continuous and intermittent administration.

**DOI:** 10.1038/bjc.1980.110

**Published:** 1980-04

**Authors:** Y. M. Peng, D. S. Alberts, H. S. Chen, N. Mason, T. E. Moon

## Abstract

We have studied the cytotoxicity of bleomycin (4--10 u/kg/day for 6 days) given by continuous i.p. infusion (using an osmotic minipump) compared to daily i.p. bolus administration, against P388 leukaemic spleen colony-forming-units(LCFU-S). Continuous i.p. bleomycin at 8 u/kg/day caused a 0.5 log greater reduction of LCFU-S than did an identical dose given by intermittent bolus administration. The infusion minipump provided constant bleomycin plasma levels of 0.62 +/- 0.03 mu/ml and a total plasma AUC (area under the plasma decay curve) of 89.0 mu.h/ml for 6 days at 8 u/kg/day. Intermittent bolus bleomycin at 8 u.kg/day had a terminal-phase plasma t1/2 of 15 min and a total 6-day plasma AUC of 90.8mu.h/ml. These pharmacokinetic data validate the osmotic minipump as a constant drug-delivery system, and suggest that the two administration schedules resulted in equal total bleomycin dosages. Although high peak bleomycin plasma levels (i.e. 32 mu/ml) were achieved with the intermittent bolus administration, continuous-infusion bleomycin's greater inhibition of LCFU-S was probably related to the drug's schedule-dependent cell-killing characteristics. The results of this study provide further rationale for the continuing use of infusion bleomycin schedules in cancer patients.


*Br. J. Cancer* (1980) **41**, 644

### ANTITUMOUR ACTIVITY AND PLASMA KINETICS OF BLEOMYCIN BY CONTINUOUS AND INTERMITTENT ADMINISTRATION

Y.-M. PENG, D. S. ALBERTS*, H.-S. G. CHEN, N. MASON AND T. E. MOON

*From the Section of Hematology and Oncology, Department of Medicine and the Cancer Center, University of Arizona College of Medicine, Tucson, Arizona* 85724

Received 22 October 1979 Accepted 11 January 1980

**Summary.—We have studied the cytotoxicity of bleomycin (4–10 u/kg/day for 6 days) given by continuous i.p. infusion (using an osmotic minipump) compared to daily i.p. bolus administration, against P388 leukaemic spleen colony-forming-units (LCFU-S). Continuous i.p. bleomycin at 8 u/kg/day caused a 0·5 log greater reduction of LCFU-S than did an identical dose given by intermittent bolus administration. The infusion minipump provided constant bleomycin plasma levels of 0·62 ± 0·03 mu/ml and a total plasma AUC (area under the plasma decay curve) of 89·0 mu.h/ml for 6 days at 8 u/kg/day. Intermittent bolus bleomycin at 8 u.kg/day had a terminal-phase plasma $t_{1/2}$ of 15 min and a total 6-day plasma AUC of 90·8mu.h/ml. These pharmacokinetic data validate the osmotic minipump as a constant drug-delivery system, and suggest that the two administration schedules resulted in equal total bleomycin dosages. Although high peak bleomycin plasma levels (*i.e.* 32 mu/ml) were achieved with the intermittent bolus administration, continuous-infusion bleomycin's greater inhibition of LCFU-S was probably related to the drug's schedule-dependent cell-killing characteristics. The results of this study provide further rationale for the continuing use of infusion bleomycin schedules in cancer patients.**

BLEOMYCIN has proven effectiveness against several human cancers when administered either intermittently (i.v., i.m., s.c. or i.p.) or by continuous infusion (Prestayko & Crooke, 1979; Alberts *et al.*, 1979). There is some evidence that continuous i.v. infusion (C.i.v.) *vs* intermittent i.v. push (I.i.v.) bleomycin (when combined with mitomycin C and vincristine) is associated with similar response rates and less systemic toxicity in patients with squamous-cell cancer of the cervix (Baker *et al.*, 1978). More recently Sikic *et al.* (1978) have shown that continuous s.c. (C.s.c.) bleomycin as compared to intermittent s.c. (I.s.c.) bleomycin caused longer survival and slower tumour growth rates and pulmonary toxicity in mice bearing Lewis lung carcinoma (Sikic *et al.*, 1978). We have studied the effect of continuous i.p. (C.i.p.) *vs* intermittent i.p. (I.i.p.) bleomycin against mouse leukaemia spleen colony growth, and have correlated the enhanced antileukaemic activity of C.i.p. with its more favourable plasma pharmacokinetics (Peng *et al.*, 1979).

### MATERIALS AND METHODS

*Mice.*—6–8-week-old male DBA/2 mice weighing ~20–25 g were purchased from the Jackson Laboratory, Bar Harbor, Maine. They were maintained on normal laboratory chow and acid water *ad. lib*.

*Mouse leukaemia.*—P388 lymphocytic leukaemia was supplied by Dr Daniel Griswold, Southern Research Institute, Birmingham, Alabama, and serially transplanted as an ascites tumour at weekly intervals ($10^6$ cells every 7 days in McCoy's 5a medium, Gibco, Grant Island, NY).

*Spleen colony assay.*—The mouse spleen-colony assay system (Alberts & Wetters,

* To whom requests for reprints should be addressed.

1976) was used to determine the efficacy of C.i.p. *vs* I.i.p. bleomycin.

On Day 0, $10^6$ P388 ascites tumour cells were injected i.v. into groups of 5 male DBA/2 mice. For the I.i.p. group, bleomycin (2–10 u/kg/day) was administered i.p. daily from Day 1 to Day 6. For the C.i.p. group, the Alzet osmotic minipump was implanted on Day 1 and was left in place for 6 days. To implant the pump, the animal was first anaesthetized with pentobarbital, a small incision was made in the abdomen, the minipump filled with bleomycin (2–10 u/kg/day) was inserted i.p. and the incision was closed with surgical clips. In addition to an untreated control group, 5 animals were implanted with minipumps containing 0·9% NaCl.

Seven days after cell injection, the mice of the untreated control and treated groups were killed, femurs isolated, and marrow cells washed out with McCoy's 5a medium. Appropriate dilutions of the leukaemic femoral marrow cells were injected i.v. into groups of 10 DBA/2 mice. Eight days later, the recipient mice were killed, spleens removed and fixed in Bouin's solution. The number of surface macroscopic, leukaemic colony-forming units (LCFU-S) were counted with a dissecting microscope, and the fraction of surviving LCFU per femur determined.

*Pharmacokinetic studies*.—On Day 0, $10^6$ P388 ascites cells were injected i.v. into groups of 30 DBA/2 mice. C.i.p. and I.i.p. bleomycin treatments were carried out as previously described. For the C.i.p. group, blood samples from at least 3 mice were collected in heparinized tubes by heart puncture every day for 6 days. For the I.i.p. group, blood samples were collected at 5, 15, 30, 45, 60, and 120 min after the administration of bleomycin on Day 5. At least 3 mice were killed at each sampling time.

Blood samples were centrifuged at 2,000 rev/min and 4°C for 10 min and the plasmas separated and immediately stored at −20°C. The bleomycin concentrations were determined using the antiserum and radioimmunoassay technique developed by Broughton & Strong (1976). Bleomycin stability in the Alzet minipump was determined at 37°C for 7 days in saline.

*Data analysis*.—Statistical analysis of the differences between LCFU dose–response (*i.e.*, drug dose *vs* fraction of CFU surviving per femur) curves for the two different treatments used the analysis of covariance method (Snedecor & Cochran, 1979).

Bleomycin plasma concentrations *vs* time data were fitted to a single exponential equation using a nonlinear regressing computer programme (Metzler, 1969). Bleomycin plasma half-lives and areas under the plasma disappearance curves (AUC) were calculated as previously described (Alberts *et al.*, 1978).

### RESULTS

Bleomycin in 0·9% NaCl had 100% stability inside the osmotic minipumps for at least 6 days at 37°C. We were therefore able to use these minipumps to deliver a constant infusion of bleomycin for 6 days.

Fig. 1 shows bleomycin dose–response curves against LCFU. C.i.p. bleomycin

FIG. 1.—Bleomycin dose–response curves against LCFU-S. The upper curve (▲) represents the inhibition of LCFU-S by daily i.p. bolus injections of bleomycin, whereas the lower curve (■) represents inhibition of LCFU-S by continuous i.p. infusion of bleomycin.

FIG. 2.—Plasma disappearance of bleomycin (8 u/kg/day) after continuous i.p. infusion (—●—) *vs* internittemt i.p. bolus (—○—) administration in adult male DBA/2 mice. Data points represent measured bleomycin concentrations by radioimmunoassay in pooled plasma samples from groups of 3 mice. Dotted lines are simulations of intermittent i.p. bolus bleomycin data obtained on Day 5.

caused an approximately 0·5 log greater reduction of LCFU than did I.i.p. bleomycin at doses of 6–10 u/kg/day ($P < 0{\cdot}01$).

The minipump provided constant bleomycin plasma levels of $0{\cdot}62 \pm 0{\cdot}03$ mu/ml and a total AUC of 89·0 mu.h/ml for 6 days at 8 u/kg/day (Fig. 2), whereas intermittent bolus bleomycin (8 u/kg/day) had a peak plasma level of 32 mu/ml (at 5 min), a terminal-phase plasma $t_{1/2}$ of 15 min and a total 6-day AUC of 90·8 mu.h/ml.

### DISCUSSION

We have shown that C.i.p. bleomycin caused a statistically greater inhibition of LCFU-S than did I.i.p. bleomycin at several points along the dose–response curve. These data and those of Sikic *et al.* (1978) showing C.s.c. bleomycin to be superior to I.s.c. bleomycin against Lewis lung carcinoma (Sikic *et al.*, 1978) and to have less pulmonary toxicity (*i.e.*, decreased content of lung collagen) strongly recommend continuous infusion as the route of choice for bleomycin administration. The study of Baker *et al.* (1978) in patients with squamous-cell carcinoma of the cervix has shown that bleomycin given by C.i.v. along with mitomycin C and vincristine had as much antitumour activity and less systemic toxicity than when the drug was given I.i.v. along with similar doses of the other 2 anticancer agents (Peng *et al.*, 1979).

Bleomycin appears to possess cell-cycle specificity in its antitumour effects (Barranco, 1979). Like cytosine arabinoside (Skipper *et al.*, 1967; Mellett, 1972) and other cell-cycle-specific agents, it may be necessary to maintain a minimal cytotoxic concentration of bleomycin while the tumour cell is traversing the S phase of the cell cycle in order to optimize cell kill. Our plasma disappearance data for bleomycin after C.i.p. administration shows that drug concentrations of greater than 0·6 mu/ml plasma are maintained for about 6 days. This is in contrast to the short periods (about 92 min) during which bleomycin plasma concentrations remain above 0·6 mu/ml after I.i.p. administration. The improved tumour-cell kill associated with C.i.p. administration in this study and C.s.c. in the study of Sikic *et al.* (1978) can probably be explained by bleomycin's more favourable plasma kinetics after continuous infusion.

Future clinical-research studies using bleomycin in the treatment of the lymphomas and testicular, head and neck and lung cancers should be designed to evaluate the efficacy of the C.i.v. route. Using C.i.v. bleomycin it may be possible to maintain or improve on I.i.v. bleomycin's antitumour activity while decreasing its pulmonary toxicity.

This work was supported by Grant CA-17094 from the National Institutes of Health, U.S. Public Health Service, Department of Health, Education, and

Welfare and The Phi Beta Sci National Sorority, Lima, Ohio.

We wish to thank Ruth Ann Lynn for her editing and typing of this manuscript.

### REFERENCES

ALBERTS, D. S. & VAN DAALEN WETTERS, T. (1976) The effect of phenobarbital on cyclosphamide antitumour activity. *Cancer Res.*, **36**, 2785.

ALBERTS, D. S., CHEN, H.-S. G., LIU, R. & 7 others (1978) Bleomycin pharmacokinetics in man. I. Intravenous administration. *Cancer Chemother. Pharmacol.*, **1**, 177.

ALBERTS, D. S., CHEN, H.-S. G., MAYERSOHN, M., PERRIER, D., MOON, T. E. & GROSS, J. F. (1979) Bleomycin pharmacokinetics in man. II. Intracavitary administration. *Cancer Chemother. Pharmacol.*, **2**, 127.

BAKER, L. H., OPIPARI, M. I., WILSON, H., BOTTOMLEY, R. & COLTMAN, C. A., JR. (1978) Mitomycin C, vincristine, and bleomycin therapy for advanced cervical cancer. *Obstet. Gynecol.*, **52**, 146.

BARRANCO, S. C. (1979) A review of the survival and cell-kinetics effects of bleomycin. In *Bleomycin: Current Status and New Developments*. Ed. Carter, Crooke & Umezawa. New York: Academic Press. p. 81.

BROUGHTON, A. & STRONG, J. E. (1976) Radioimmunoassay of bleomycin. *Cancer Res.*, **36**, 1418.

MELLETT, L. B. (1973) Considerations in design of optimal therapeutic schedules, Pharmacology and the Future of Man. In *Problems of Therapy*, Vol. 3. Ed. Okita & Acheson. Basel: S. Karger. p. 332.

METZLER, C. M. (1969) *Technical Report* 7292/69/7292/005. Kalamazoo, MI: Upjohn Co.

PENG, Y. M., ALBERTS, D. S. & WOOD, D. A. (1979) Antitumour effects and pharmacokinetics of continuous infusion (Con) *vs* intermittent (Int) bleomycin (Bleo). *Proc. AACR and ASCO*, **20**, 202.

PRESTAYKO, A. W. & CROOKE, S. T. (1979) Clinical pharmacology of bleomycin. In *Bleomycin: Current Status and New Developments*. Eds. Carter, Crooke & Umezawa. New York: Academic Press. p. 117.

SIKIC, B. I., COLLINS, J. M., MIMNAUGH, E. G. & GRAM, T. E. (1978) Improved therapeutic index of bleomycin when administered by continuous infusion in mice. *Cancer Treat. Rep.*, **62**, 2011.

SKIPPER, H. E., SCHABEL, F. M., JR & WILCOX, W. S. (1967) Experimental evaluation of potential anticancer agents. XXI. Scheduling of arabinosyl cytosine to take advantage of its S-phase specificity against leukaemia cells. *Cancer Chemother Rep.*, **51**, 125.

SNEDECOR, G. W. & COCHRAN, W. G. (Eds) (1979) *Statistical Methods*, 6th Ed. Ames: Iowa State University Press. p. 342.